# Hybrid inhibitors of DNA and HDACs remarkably enhance cytotoxicity in leukaemia cells

**DOI:** 10.1080/14756366.2020.1754812

**Published:** 2020-04-21

**Authors:** Yoojin Song, Sun You Park, Zhexue Wu, Kwang-Hyeon Liu, Young Ho Seo

**Affiliations:** a College of Pharmacy, Keimyung University, Daegu, Republic of Korea; b BK21 Plus KNU Multi-Omics based Creative Drug Research Team, College of Pharmacy and Research Institute of Pharmaceutical Sciences, Kyungpook National University, Daegu, South Korea

**Keywords:** DNA damage, histone deacetylases, cancer, leukaemia

## Abstract

Chlorambucil is a nitrogen mustard-based DNA alkylating drug, which is widely used as a front-line treatment of chronic lymphocytic leukaemia (CLL). Despite its widespread application and success for the initial treatment of leukaemia, a majority of patients eventually develop acquired resistance to chlorambucil. In this regard, we have designed and synthesised a novel hybrid molecule, chloram-HDi that simultaneously impairs DNA and HDAC enzymes. Chloram-HDi efficiently inhibits the proliferation of HL-60 and U937 leukaemia cells with GI_50_ values of 1.24 µM and 1.75 µM, whereas chlorambucil exhibits GI_50_ values of 21.1 µM and 37.7 µM against HL-60 and U937 leukaemia cells, respectively. The mechanism behind its remarkably enhanced cytotoxicity is that chloram-HDi not only causes a significant DNA damage of leukaemia cells but also downregulates DNA repair protein, Rad52, resulting in the escalation of its DNA-damaging effect. Furthermore, chloram-HDi inhibits HDAC enzymes to induce the acetylation of *α*-tubulin and histone H3.

## 1. Introduction

**Figure 1.**
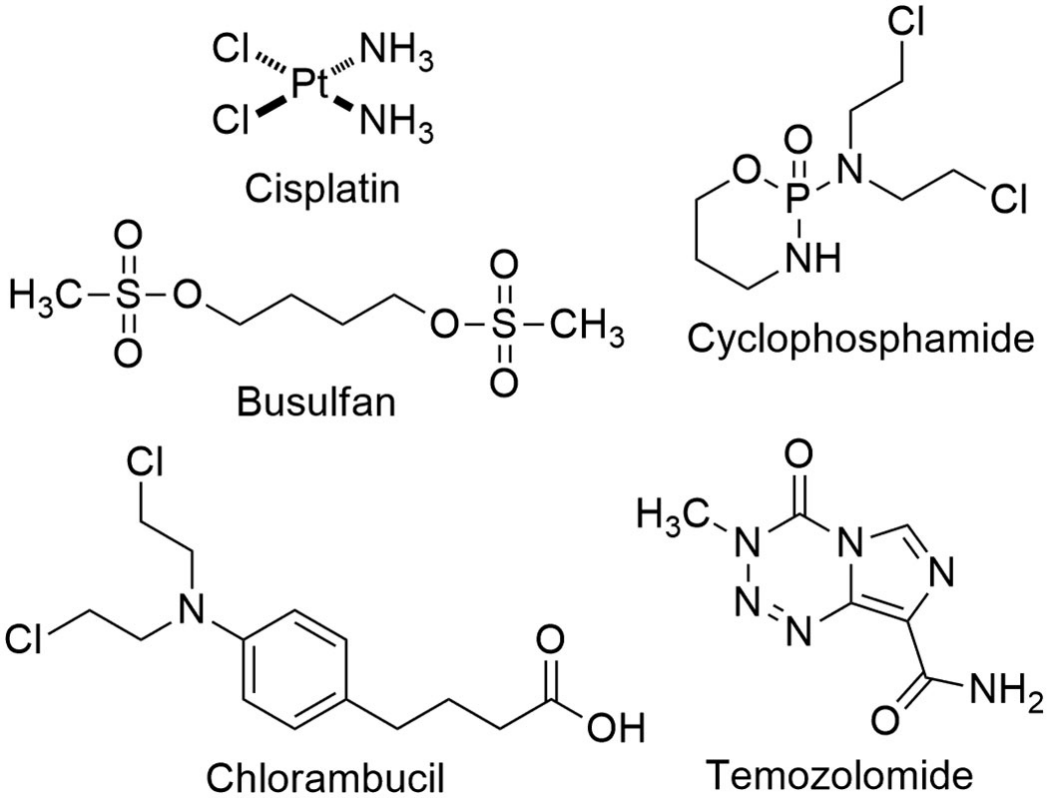
Structure of representative genotoxic drugs.

Genotoxic drugs represent an important class of chemotherapy and constitute a major treatment modality of human cancers. Genotoxic drugs such as cisplatin, busulfan, cyclophosphamide, chlorambucil, and temozolomide, cause various types of DNA damage, exerting their anticancer activity (Figure 1). Among many different DNA lesions resulting from genotoxic drugs, double-strand breaks (DSBs) are the most deleterious and a single irreparable DSB is sufficient to induce apoptosis^1^. Despite such potent lethality of genotoxic drugs, cancer cells often mitigate DNA damage by their intrinsic DNA repair capability, render drug resistance and ultimately lead to treatment failure. DNA DSBs are repaired by two distinct pathways, non-homologous end joining (NHEJ) and homologous recombination (HR). NHEJ allows for direct ligation of the DSB ends in the G_0_/G_1_ phase of the cell cycle, involving KU70/80, DNAPK and DNA ligase IV, while HR utilises homologous DNA sequences as a template accurately to restore the genomic sequence in the S/G_2_ phase of the cell cycle, mediated by BRCA1, BRCA2 and Rad52^2^^,^^3^. In eukaryotic cells, DNA damage triggers a sophisticated network of DNA damage response (DDR), which includes a sensing of DNA damage, the assembly of DNA repair factors, cell cycle transit arrest, and the activation of DNA repair for maintaining genomic integrity^4^^,^^5^. Conversely, dysregulation of DDR causes genomic instability, leading to activation of cell death pathway. The physiological importance of DDR is clearly illustrated by the severe cancer susceptibility of inherited defects in DDR factors, such as BRCA1 and BRCA2^6–8^. Consequently, drugs that interfere DNA damage response (DDR) network, such as PARP1, ATM, and DNAPK, have been intensely investigated, resulting in the first clinical approval of PARP1 inhibitor, olaparib (tradename; Lynparza) as an anticancer drug in 2014^9^^,^^10^.

Chlorambucil is a bifunctional DNA alkylating drug, belonging to a member of the nitrogen mustard. First synthesised in 1953, chlorambucil still remains one of the front-line treatment of chronic lymphocytic leukaemia (CLL) and malignant lymphomas^11–13^. The nitrogen mustards share a common mechanism of action stemming from the presence of *N*,*N*-bis(2-chloroehtyl)-amine moiety. Owing to the high electrophilic reactivity of the nitrogen mustard moiety, chlorambucil readily reacts with separate DNA bases, forms DNA interstrand crosslinks and causes DSBs^14^. In this regard, exposure of chlorambucil promotes apoptotic cell death of human cancers via the accumulation of persistent DNA damage.

Although approximately 60–80% of patients respond to chlormabucil for years, eventually all patients become resistant to this drug^11^^,^^15^. The mechanism of chlorambucil resistance is poorly understood. However, there are several reports suggesting that DNA repair system including homologous recombination (HR) are the major culprit to deactivate its therapeutic potency^15–17^. Accordingly, the occurrence of drug resistance is a serious impediment to the successful treatment of chronic lymphocytic leukaemia (CLL) and malignant lymphomas with chlorambucil.

**Figure 2.**
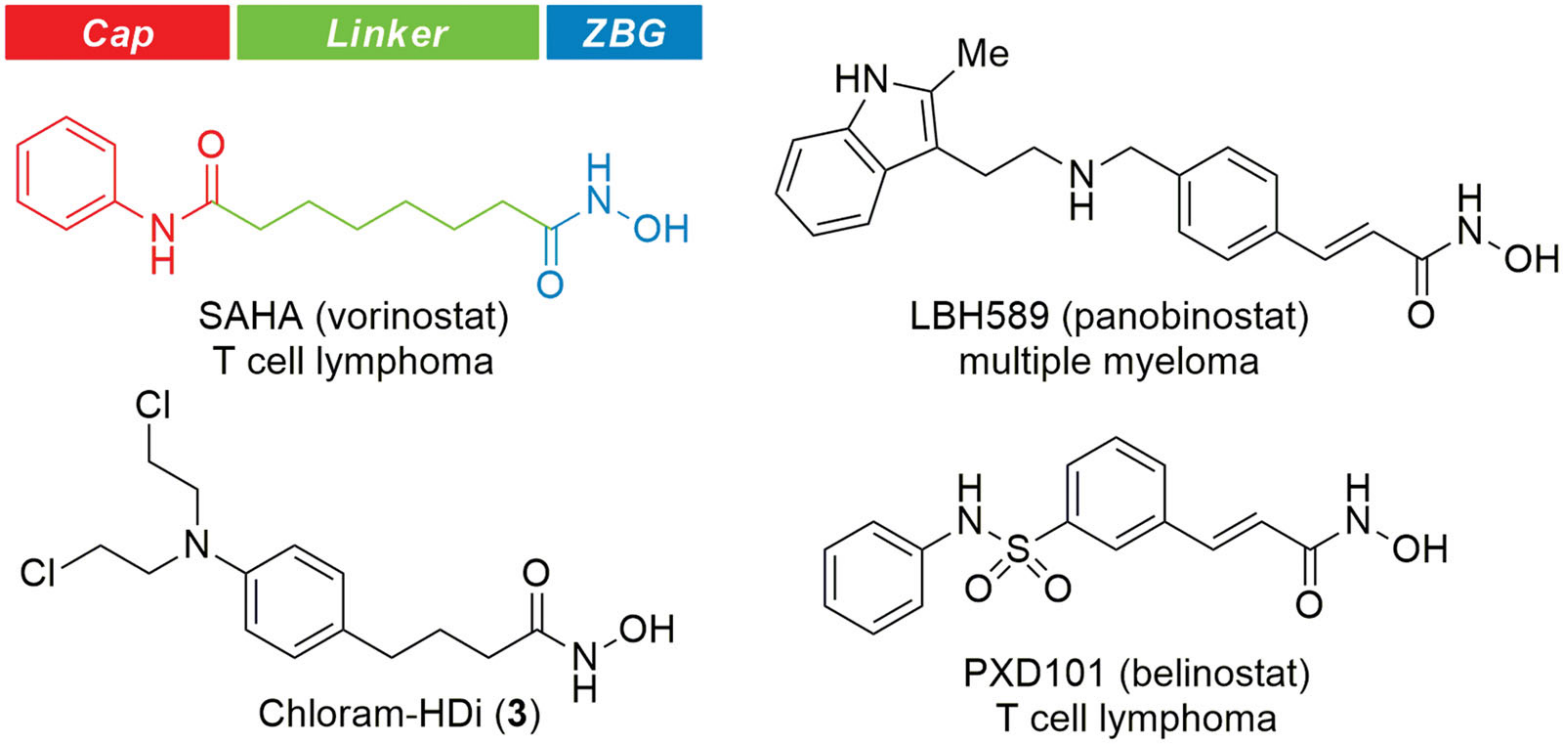
Structure of HDAC inhibitors.

Histone deacetylases (HDACs) are enzymes responsible for catalysing the removal of acetyl groups from lysine residues on histone, leading to gene transcription or silencing^18^. Apart from histone modification, HDACs also regulate the deacetylation of non-histone proteins, including transcription factors, chaperones, signalling molecules, and DNA repair proteins^19–21^. In recent years, inhibition of HDACs has emerged as a promising therapeutic target for cancers and several other diseases^22–26^. As a result, significant efforts have been made to identify HDAC inhibitors, leading to the approval of SAHA (vorinostat), FK228 (romidepsin), LBH589 (panobinostat), and PXD101 (belinostat) as anticancer drugs (Figure 2)^27–36^.

HDAC inhibitors are grouped structurally into four classes, hydroxamic acids, cyclic tetrapeptides, benzamides, and short-chain fatty acids^20^^,^^21^. Most classes of HDAC inhibitors contain the common pharmacophore, which is composed of three distinct domains, a zinc-binding domain, a linker domain, and a cap domain^37^^,^^38^. The zinc-binding domain chelates the catalytic Zn^2+^ ion in the active site, which is critical for catalytic function of HDACs, the cap domain is a surface recognition group that interacts with the entrance of the active site pocket, and the linker domain connects the cap domain to the zinc-binding domain.

## 2. Results and discussion

### 2.1. Drug design and synthesis

Recently, it has been reported that the inhibition of HDAC enzymes suppress DNA repair machinery and sensitises cancers to genotoxic drugs^39^^,^^40^. Based on this, we determined to develop small molecule inhibitors, which could simultaneously attack DNA and HDACs. To this end, a novel hybrid, chloram-HDi (**3**) was designed by coupling chlorambucil with hydroxamic acid as a zinc-binding group, anticipating that chloram-HDi (**3**) could inhibit HDACs function by chelating the catalytic Zn^2+^ ion in the active site of HDACs without losing the inherent DNA damaging capability of chlrambucil. We hypothesised that chloram-HDi (**3**) impaired DNA HR repair system via inactivation of HDACs, while nitrogen mustard moiety of **3** caused DSBs by alkylating DNA, and accumulated DNA damage. Besides, the fact that DNA and HDACs mostly exist in a same nuclear compartment, makes them more prone to dual inhibition by chloram-HDi (**3**), circumventing difficult pharmacokinetics of dual targeting. During our investigation of novel hybrid DNA-HDACs inhibitors, Yuan’s group reported a DNA-HDAC inhibitor by attaching a benzamide zinc-binding group to chlorambucil. However, their inhibitor did not show good HDACs inhibitory activities as well as potent anti-proliferative activities against cancer cell lines^41^.

**Scheme 1.**
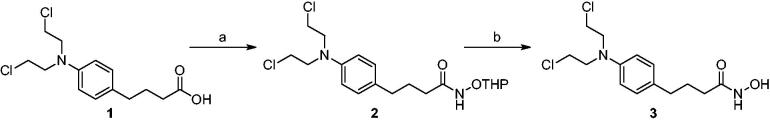
Synthesis of compound **3**. Reagents and conditions: (a) NH_2_OTHP, EDC, DIPEA, DMF, rt, 12 h; (b) 1 *N* HCl, CH_2_Cl_2,_ rt, 1 h, 34% (2 steps).

We first pursued the synthesis of chloram-HDi (**3**) from chlorambucil (**1**), which was equipped with hydroxamic acid moiety as zinc-binding group (ZBG), as shown in Scheme 1. To do so, amide coupling reaction of chlorambucil (**1**) with NH_2_OTHP was carried out in the presence of EDC, HOBt, and DIPEA in DMF to afford compound **2**. Subsequent cleavage of THP group with 1 *N* HCl successfully provided compound **3** in 34% yield for two steps.

**Scheme 2.**
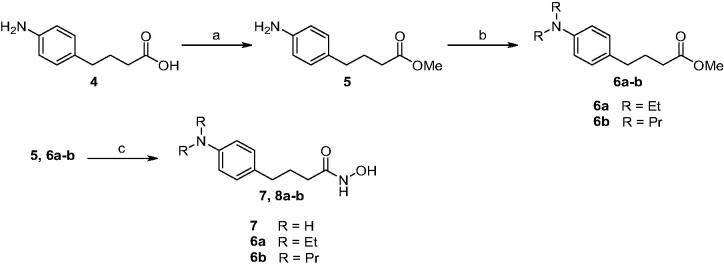
Synthesis of compound **7** and **8a-b**. Reagents and conditions: (a) H_2_SO_4_, MeOH, rt, 12 h, 86%; (b) Ethyl iodide for **6a** or propyl iodide for **6b**, K_2_CO_3,_ DMF, rt, 24 h, 75-76%; (c) NH_2_OH, KOH, MeOH, 0 °C, 3 h, 40-46%.

We next embarked on synthesis of compound **7** and **8a-b**, which did not have electrophilic chloro group unlike chloram-HDi (Scheme 2). The synthesis of **7** and **8a-b** commenced with the esterification of carboxylic acid **4** in the presence of sulphuric acid in methanol to give ester **5** in 86% yield. Methyl Ester **5** was then alkylated with ethyl iodide or propyl iodide to afford **6a-b** in 75–76% yield. Finally, methyl ester **5** and **6a-b** were treated with hydroxylamine in the presence of potassium hydroxide in methanol to afford **7** and **8a-b** in 40–46% yield.

### 2.3. Biological evaluations of compounds

Upon completion of synthesis, compound **1**, **3**, **7**, and **8a-b** were evaluated for their anti-proliferative activity against various cancer cell lines, including human acute myeloid leukaemia (AML) cell lines (U-937 and HL-60), human breast cancer cell lines (MDA-MB-231 and MCF-7), and a human ovarian cancer cell line (A2780). Each cancer cell line was treated with the indicated compound for 3 days and its anti-proliferative effect on cancer cell lines was measure using MTS colorimetric assay. As shown in Table 1, chloram-HDi (**3**) displayed greater anti-proliferative activities than its parent drug, chlorambucil (**1**). Interestingly, chloram-HDi exerted the most potent anti-proliferative activity against AML cell lines, U-937 and HL-60 with GI_50_ values of 1.75 µM and 1.24 µM respectively, which were 17–21 fold lower than GI_50_ values of chlorambucil. In contrast, chloram-HDi showed relatively poor anti-proliferative activity against human breast cancer cell lines, MDA-MB-231 and MCF-7, in that its GI_50_ values against MDA-MB-231 and MCF-7 were 95.9 µM and 244.9 µM, respectively. Compound **7** and **8a-b**, which are lack of nitrogen mustard moiety furnished either poor or mediocre anti-proliferative activities against tested cancer cell lines, indicating that nitrogen mustard moiety is an essential warhead in this anticancer drug design.

**Table 1.** Growth inhibition of compound **1**, **3**, **7**, and **8a-b** in various cancer cell lines
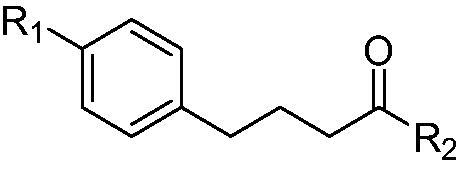

Compound	R_1_	R_2_	GI_50_ values (μM)				
			U-937	HL-60	MDA-MB-231	MCF-7	A2780
Chlorambucil (**1**)		OH	37.7	21.1	>100	393	>100
Chloram-HDi (**3**)		NHOH	1.75	1.24	95.9	244.91	21.2
**7**		NHOH	93.0	60.5	>100	393	>100
**8a**		NHOH	>100	70.3	85.1	282	81
**8b**		NHOH	40.2	34.4	66.8	37.8	7.79

**Figure 3.**
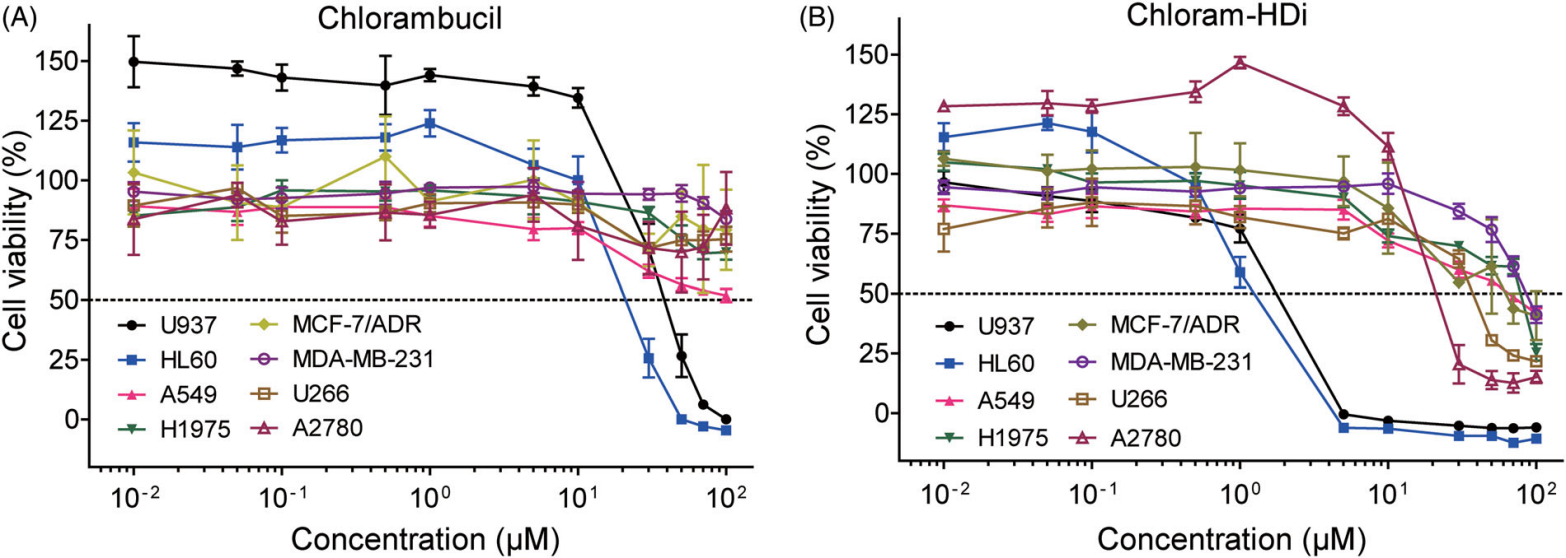
Effects of (A) chlorambucil and (B) chloram-HDi on the growth inhibition of various cancer cell lines. Cells were incubated with various concentrations of chlorambucil or chloram-HDi for 3 days, and cell proliferation was measured using the MTS assay. Data are presented as mean ± SD (*n* = 4). (C) GI_50_ values of chlorambucil and chloram-HDi against various cancer cell lines.

We next determined the effect of chlorambucil and chloram-HDi on the growth inhibition of various cancer cell lines (HL60, U937, acute myeloid leukaemia; A549, non-small cell lung carcinoma; H1975, gefitinib-resistant non-small cell lung carcinoma (NSCLC); MCF-7/ADR, multidrug-resistant breast adenocarcinoma; MDA-MD-231, mammary carcinoma; U266, multiple myeloma; A2780, ovarian carcinoma). As shown in Figure 3, chloram-HDi displayed significantly enhanced anti-proliferative activities against various cancer cell lines, compared to its parent drug, chlorambucil. Especially, the GI_50_ values (concentration in 50% growth inhibition) of chloram-HDi (1.24 µM and 1.75 µM) against HL-60 and U937 cancer cell lines were obviously much lower than those of chlorambucil (21.1 µM and 37.7 µM), indicating that chloram-HDi was superior to the clinical drug, chlorambucil in cellular potency (Table 2).

**Table 2.** GI_50_ values of chlorambucil and chloram-HDi against various cancer cell lines

Cancer cell lines	Chlorambucil (GI_50_; μM)^a^	Chloram-HDi (GI_50_; μM)
HL60	21.1	1.24
U937	37.7	1.75
A549	>100	65.3
H1975	>100	76.7
MCF-7/ADR	>100	62.1
MDA-MB-231	>100	83.5
U266	>100	38.1
A2780	>100	21.2

^a^GI_50_ values were obtained after the treatment of cells for 72 h.

**Figure 4.**
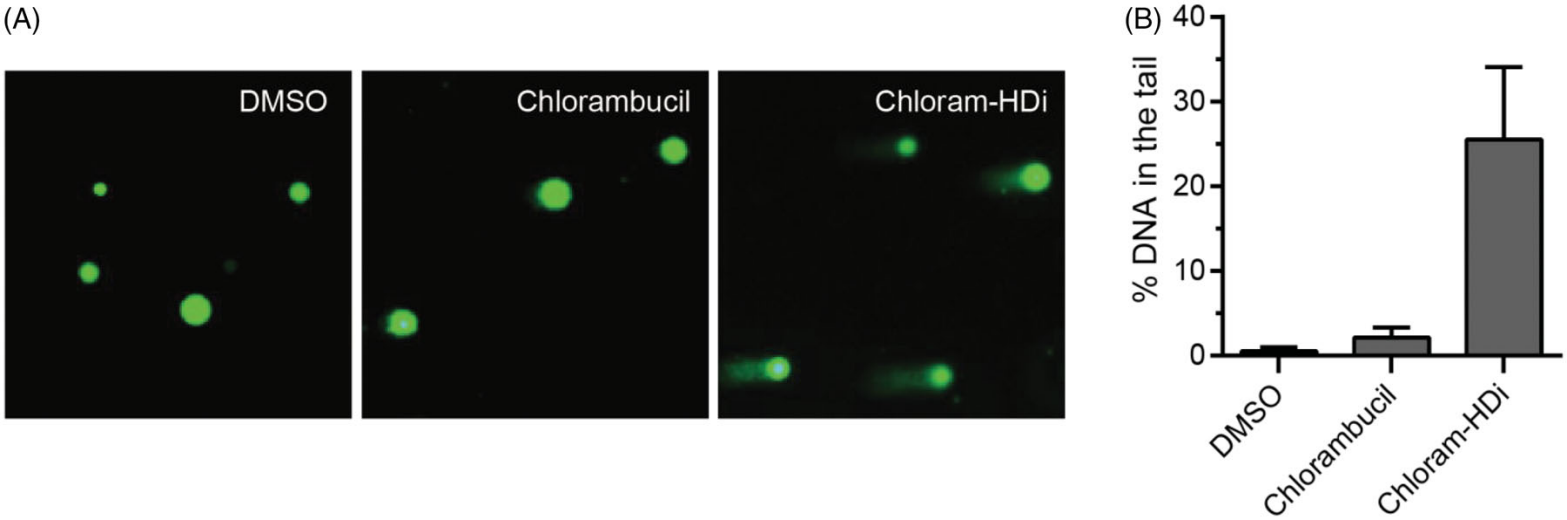
Effect of chloram-HDi on DNA damage in HL-60 cells. (A) Fluorescent images of DNA from chlorambucil or chloram-HDi treated HL-60 cells in comet assay. HL-60 cells were treated with chlorambucil (10 μM) and chloram-HDi (10 μM) for 24 h and then subjected to comet assay. DMSO is used as a negative control. (B) Comet assay results are graphed as median % DNA in the tail ± SD (*n* = 4).

The comet assay is a sensitive and rapid technique for determining the amount of DNA damage in a single cell^42^. This assay directly allows the microscopic observation of the “comet tail” that is associated with the damaged DNA content. Therefore, we investigated the impact of chloram-HDi and chlorambucil on the integrity of DNA in HL-60 cells using the comet assay (Figure 4). We first treated HL-60 cells with chloram-HDi or chlorambucil for 24 h and then measured DNA damage using the comet assay. The assay indicated that chloram-HDi (10 µM) caused more noticeable DNA damage than chlorambucil (10 µM), in that % DNA content in the tail induced by chloram-HDi and chlorambucil were 25.5% and 2.1%, respectively (Figure 2(B)).

**Figure 5.**
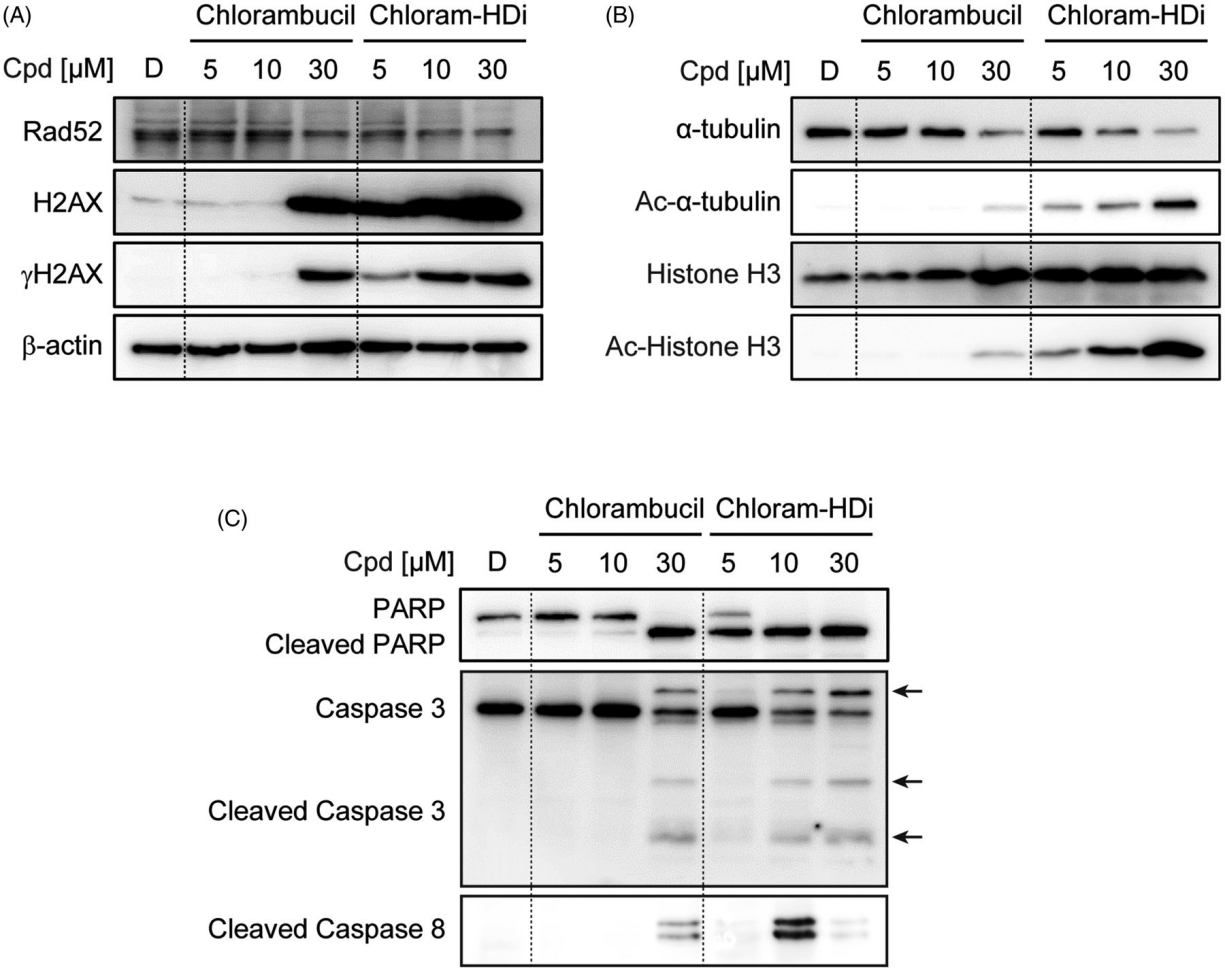
Treatment of HL-60 cells with chloram-HDi enhances DNA damage and induces apoptosis of HL-60 cancer cells. (A) Effect of chloram-HDi on the DNA damage markers, Rad52, H2AX, and *γ*-H2AX. HL-60 cells were treated with chlorambucil (10 μM) and chloram-HDi (10 μM) for 24 h and then subjected to western blot analysis. (B) Chloram-HDi promotes the acetylation of *α*-tubulin and Histone H3 though inhibition of HDAC6 and HDAC1. (C) Chloram-HDi induces the cleavage of PARP, caspase 3, and caspase 8.

We next investigated the precise mechanism of action behind the increased DNA-damaging effect of chloram-HDi compared with its parent drug, chlorambucil. Hence, we first treated HL-60 cells with the indicated concentrations of chlorambucil and chloram-HDi for 24 h and measured the expression levels of Rad52, H2AX, *γ*-H2AX, and *β*-actin. *γ*-H2AX is a well- established hallmark of DNA double-strand breaks and Rad52 is an important protein in the homologous recombination repair (HRR) of DNA double-strand breaks^43–45^. As shown in Figure 5(A), the treatment of cells with chloram-HDi more significantly increased the expression of *γ*-H2AX than chlorambucil in a dose-dependent manner, indicating that chloram-HDi effectively induced more DNA damage than chlorambucil, which was consistent with the result that we observed in the comet assay. Furthermore, chlrom-HDi downregulated the protein level of Rad52 in a dose-dependent manner. It has been reported that the inhibition of HDACs leads to the reduction of Rad52 protein level. Nevertheless, the reduction of the DNA repair protein, Rad52 contributed to the increased DNA-damaging effect of chloram-HDi, offering a distinct advantage over chlorambucil.

To assess the comparative effect of chlorambucil and chloram-HDi on the acetylation status of *α*-tubulin and histone H3, we also measured the expression levels of *α*-tubulin, Ac-*α*-tubulin, histone H3, and Ac-histone H3 (Figure 5(B)). *α*-Tubulin and histone H3 are well documented substrates of HDAC6 and HDAC1^46^^,^^47^. Accordingly, the inhibition of HDAC6 and HDAC1 enzymes epigenetically induced the acetylation of *α*-tubulin and histone H3, respectively. The treatment of HL-60 cells with chloram-HDi dose-dependently increased the acetylation of *α*-tubulin and histone H3, while total amount of histone H3 remained unchanged. It is interesting to note that chloram-HDi decreased the expression level of *α*-tubulin in a dose-dependent manner. In contrast, the treatment of HL-60 cells with chlorambucil (5 and 10 µM) failed to induce the acetylation of *α*-tubulin and histone H3, whereas 30 µM of chlorambucil resulted in little acetylation of *α*-tubulin and histone H3. Taken together, the result undoubtedly demonstrated that chloram-HDi efficiently inhibited the function of HDAC6 and HDAC1 enzymes and promoted the cellular acetylation status of *α*-tubulin and histone H3.

The activation of apoptotic signals can suppress the proliferation of cancer cells, as it is regulated by the avoidance of the apoptotic mechanism. To determine if the anti-proliferative effect of chloram-HDi on HL-60 cells is associated with the induction of apoptosis, we therefore measured the cleavage of apoptotic biomarkers, including PARP, caspase 3, and caspase 8. As shown in Figure 5(C), the exposure of HL-60 cells with chloram-HDi significantly promoted the cleavage of PARP, caspase 3, and caspase 8 in a dose-dependent manner. Conversely, when HL-60 cells were treated with chlorambucil, the administration of 30 µM concentration was required to induce the cleavage of PARP, caspase 3, and caspase 8, clearly indicating that chloram-HDi more potently led to the apoptotic cancer cells death than chlorambucil.

**Figure 6.**
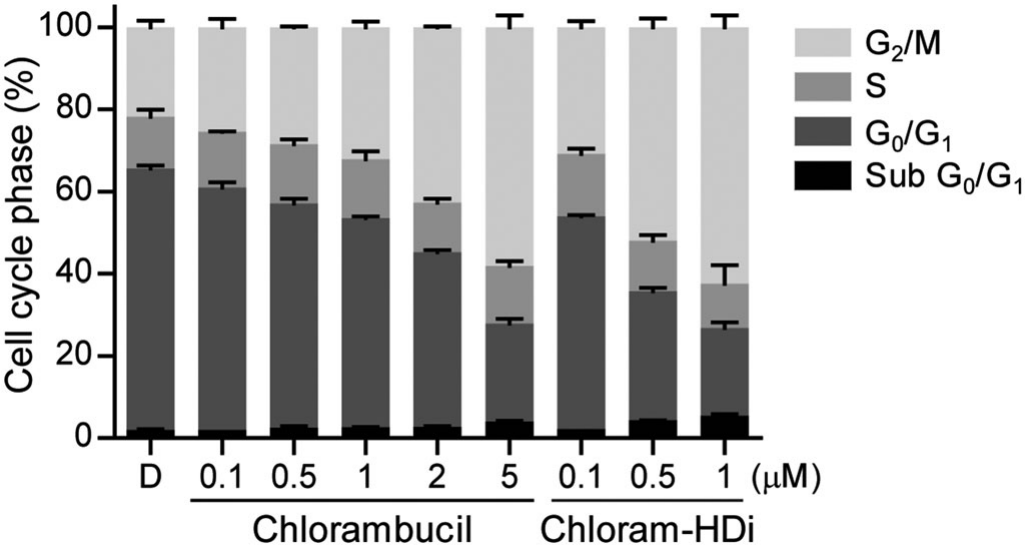
Chloram-HDi induces G_2_/M cell cycle arrest. The effect of chlorambucil and chloram-HDi on the cell cycle was analysed by flow cytometry. HL-60 cells were treated with the indicated concentrations of chlorambucil and chloram-HDi for 24 h. HL-60 cells were stained with propidium iodide and cell cycle distribution was analysed by flow cytometry. DMSO (D) was used as a negative control.

To determine whether chloram-HDi affected the cell cycle, we evaluated the effect of chloram-HDi on the cell cycle distribution of HL-60 cells using flow cytometry. After exposed to the indicated concentrations of chlorambucil and chloram-HDi for 24 h, the cell cycle distribution of HL-60 cells was measured. As shown in Figure 6, the exposure of HL-60 cells with chloram-HDi remarkably promoted the cell cycle arrest in the G_2_/M phases, compared with the DMSO control and the reference drug chlorambucil in that the prominence of G_2_/M cell cycle arrest is a reported feature of the nitrogen mustards^48^^,^^49^. The result indicated that chloram-HDi caused the DNA double-strand breaks of HL-60 cells, leading to the activation of the homologous recombination repair (HRR) pathway through G_2_/M cell cycle arrest. Conversely, another DNA DSB repair pathway, Non-homologous end joining (NHEJ) is reported to primarily occur in G_0_/G_1_ phase^2^. The treatment of HL-60 cells with 0.1 µM of chloram-HDi increased G_2_/M fraction from 21.8% to 30.8%, while the treatment of HL-60 cells with 1 µM of chlorambucil led to 32.2% G_2_/M arrest of HL-60 cells, which indicated that chloram-HDi was more efficacious than chlorambucil in the cell cycle arrest. When the cells were treated with the increased concentration of chloram-HDi, the G_2_/M arrest was more remarkable (52% G_2_/M fraction at 0.5 µM and 62.4% G_2_/M fraction at 1 µM). Conversely, the treatment of cells with chloram-HDi resulted in a reduction of the cell population at the G_0_/G_1_ phases in a dose-dependent manner. It is also interesting to note that chloram-HDi increased the cell population at sub G_0_/G_1_ phases in a dose-dependent manner. Taken together, the results suggested that chloram-HDi effectively induced the cell cycle arrest at the G_2_/M phases in HL-60 cells in a dose-dependent manner.

We next investigated the inhibitory activity of chloram-HDi against HDAC1, 3, 6, and 7 isoforms, in that the clinically approved HDAC inhibitor, SAHA was used as a reference drug (Table 3). Although chloram-HDi displayed less efficacious inhibitory activities than the reference drug SAHA against all tested HDACs enzymes, chloram-HDi still displayed modest inhibitory activities against HDACs enzymes. Interestingly, chloram-HDi more selectively inhibited HDAC6 enzyme with an IC_50_ value of 694 nM than other HDAC enzymes (IC_50_ of HDAC1=4.497 µM, IC_50_ of HDAC3=6.201 µM, and IC_50_ of HDAC7=74.568 µM).

**Table 3.** IC_50_ values of chloram-HDi against HDAC enzymes

Class	Enzymes	IC_50_ values (nM)	
		Chloram-HDi	SAHA
I	HDAC1	4,497	219
	HDAC3	6,201	107
IIb	HDAC6	694	49
IIa	HDAC7	74,568	61,020

**Figure 7.**
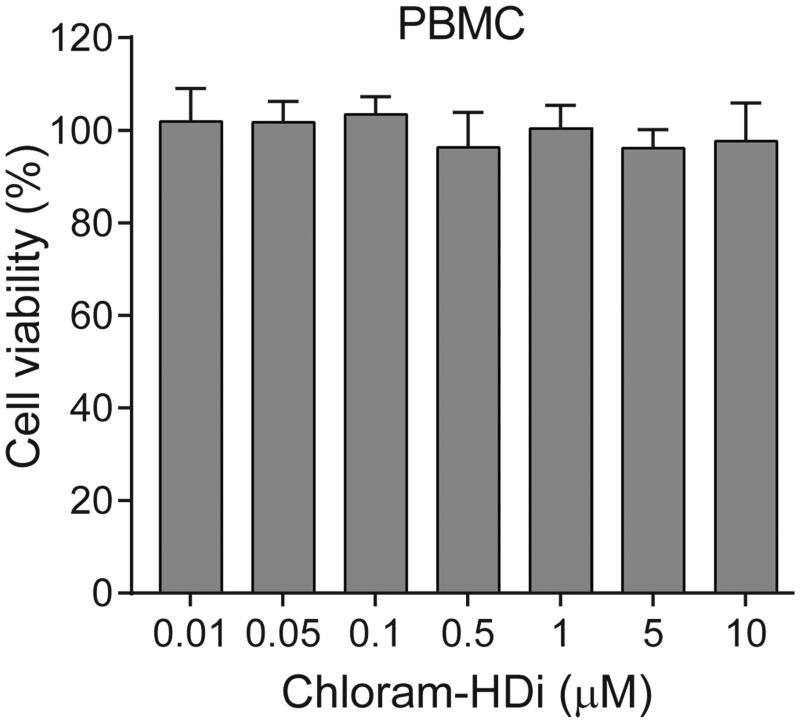
Effect of chloram-HDi on the cell viability of PBMCs. Cells were incubated with chloram-HDi (0.01, 0.05, 0.1, 0.5, 1, 5, and 10 μM) for 3 days and cell viability was measured using the colorimetric MTS assay. Data are presented as mean ± SD (*n* = 4).

To determine whether chloram-HDi was toxic to the normal cells, we isolated primary human peripheral blood mononuclear cells (PBMCs) and examined the cytotoxicity of chloram-HDi on normal human PBMCs. Cells were first treated with various concentrations of chloram-HDi for 3 days and cell viability was measured using the colorimetric MTS assay. As shown in Figure 7, the result indicated that chloram-HDi was not toxic to normal human PBMCs up to 10 µM concentration, suggesting that chloram-HDi selectively targets the malignant leukaemia cell lines HL-60 (IC_50_ = 1.24 µM) and U937 (IC_50_ = 1.75 µM) over the normal PBMCs (IC_50_ > 10 µM).

Cytochrome P450 (P450) enzymes have emerged as an important determinant in the occurrence of drug interactions that can lead to adverse drug reactions. Therefore, we investigated the effect of chloram-HDi (**3**) on the catalytic activities of clinically significant human P450s such as 1A2, 2C9, 2C19, 2D6, and 3 A in human liver microsomes^50^^,^^51^. To do so, the inhibitory potency of chloram-HDi (**3**) was determined with cytochrome P450 assays in the absence and the presence of chloram-HDi (**3**) up to 50 µM final concentration using pooled human liver microsomes (Table 4). The assay indicated that chloram-HDi (**3**) showed weak inhibitory effect (>50 µM) against five P450 isoforms. These findings suggest that clinical interactions between chloram-HDi (**3**) and substrate drugs of five P450 isoforms would not be expected.

**Table 4.** Inhibitory potency of chloram-HDi on specific P450 activities in human liver microsomes

Enzyme activity	P450	IC_50_ (μM)
Phenacetin *O*-deethylation	1A2	>50
Tolbutamide 4-methylhydroxylation	2C9	>50
Omepraozle hydroxylation	2C19	>50
Dextromethorphan *O*-demethylation	2D6	>50
Midazolam 1′-hydroxylation	3A	>50

### 2.3. Molecular modelling of chloram-HDi (3)

**Figure 8.**
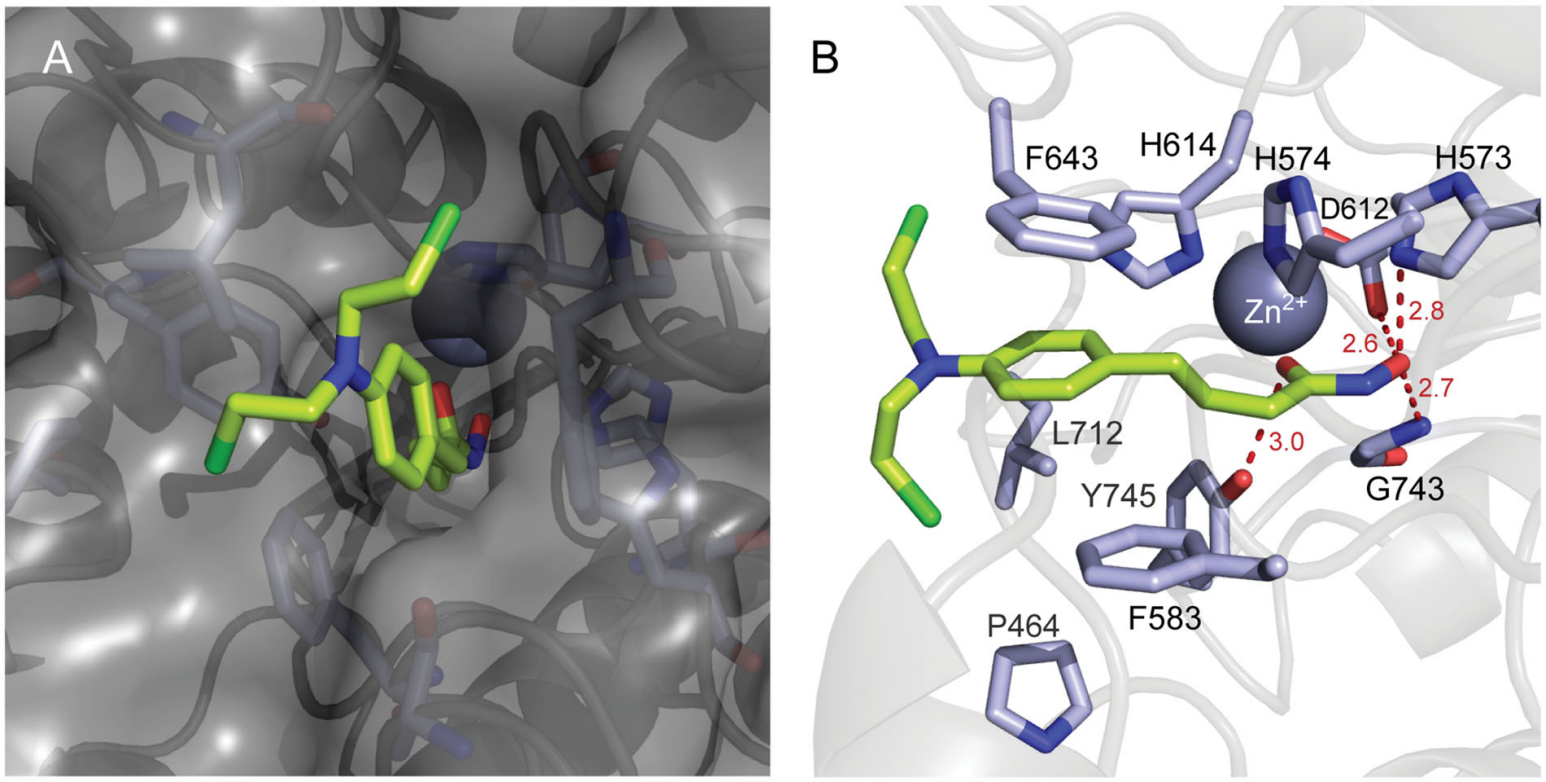
Molecular docking model of chloram-HDi (**3**) bound in the substrate-binding pocket of HDAC6 (PBD code: 5EF7). (A) Surface representation of HDAC6 and chloram-HDi (**3**) complex. (B) Cartoon and sticks representation of HDAC6 and chloram-HDi (**3**) complex. The carbon, oxygen, nitrogen and chlorine atoms of chloram-HDi (**3**) are shown in lime, red, blue, and green, respectively. The side chains of the substrate-binding site are coloured according to the atom types (carbon, light blue; oxygen, red; nitrogen, blue) and labelled with their residue name. The hydrogen bonds are shown as dashed red lines. The docking poses are visualised using PyMOL1.3.

To investigate the binding mode of chloram-HDi (**3**), *in silico* docking studies were performed with HDAC6 enzymes (Figure 8). Modelling chloram-HDi (**3**) in the substrate-binding pocket of HDAC6 (PBD code: 5EF7) indicated that chloram-HDi (**3**) effectively bound into a deep substrate-binding pocket of HDAC6 (Figure 8(A)). The middle phenylpropyl group of chloram-HDi (**3**) well occupied the lipophilic channel and formed favourable π-π stacking interactions with F643 and F583 residues (Figure 8(B)). The hydroxamate C=O and OH groups of chloram-HDi (**3**) chelated the active site Zn^2+^ ion in a bidentate manner and formed additional hydrogen bond interactions with Y745, D612, H573, and G743 residues. In contrast, the nitrogen mustard moiety of chloram-HDi (**3**) was located in the rim of the substrate-binding pocket, participating in Van der Waals interactions with the hydrophobic patches of the rim, composed of F643 and F583 residues.

## 3. Experimental

### 3.1. Chemistry

#### 3.1.1. General methods and materials

Unless otherwise noted, all reactions were performed under argon atmosphere in oven-dried glassware. All purchased reagents and solvents were used without further purification. Thin layer chromatography (TLC) was carried out using Merck silica gel 60 F_254_ plates. TLC plates were visualised using a combination of UV light and iodine staining. Column chromatography was conducted under medium pressure on silica (Merck Silica Gel 40–63 µm) or performed by MPLC (Biotage Isolera One instrument) on a silica column (Biotage SNAP HP-Sil) or C18 column (Biotage SNAP Ultra C18). NMR analyses were carried out using a JNM-ECZ500R (500 MHz) manufactured by Jeol resonance. ^1^H and ^13 ^C NMR chemical shifts are reported in parts per million (ppm). The deuterium lock signal of the sample solvent was used as a reference, and coupling constants (*J*) are given in hertz (Hz). The splitting pattern abbreviations are as follows: s, singlet; d, doublet; t, triplet; q, quartette; dd, doublet of doublets; m, multiplet. The purity of all tested compounds was confirmed to be higher than 95% by HPLC analysis performed with a dual pump Shimadzu LC-6AD system equipped with VP-ODS C18 column (4.6 mm × 250 mm, 5 µm, Shimadzu).

#### 3.1.2. 4–(4-(Bis(2-chloroethyl)amino)phenyl)-N-hydroxybutanamide (3)

A mixture of chlorambucil (0.30 g, 1.00 mmol), NH_2_OTHP (0.13 g, 1.10 mmol), 1-ethyl-3–(3-dimethylaminopropyl)carbodiimide (0.28 g, 1.48 mmol) and *N,N*-diisopropylethylamine (0.35 ml, 1.97 mmol) in DMF (5 ml) was stirred at room temperature for 12 h under argon. The solution was diluted with ethyl acetate and washed with brine. The organic layer was dried over Na_2_SO_4_, filtered and concentrated under reduced pressure. The resulting intermediate was dissolved in CH_2_Cl_2_ (8 ml) and treated with 1 *N* HCl in Et_2_O (8 ml) at room temperature for 1 h. The precipitating solid was filtered off and purified by MPLC (Biotage SNAP KP-C18 column) to afford compound **3** in 34% yield. R_f_ = 0.26 (7:3 ethyl acetate: hexanes). ^1^H NMR (500 MHz, MeOD) δ 7.05 (d, *J*=8.5 Hz, 2 H), 6.68 (d, *J*=8.0 Hz, 2 H), 3.72 (t, *J*=6.5 Hz, 4 H), 3.51 (t, *J*=6.5 Hz, 4 H), 2.52 (t, *J*=7.5 Hz, 2 H), 2.30 (t, *J*=7.5 Hz, 2 H), 1.89–1.83 (m, 2 H). ^13 ^C NMR (125 MHz, MeOD) δ 172.8, 145.9, 131.5, 13.5, 113.4, 54.5, 48.4, 35.0, 33.1, 28.8 ESI MS (*m/z*) 320.25 [M + 1]^+^.

#### 3.1.3. Methyl 4–(4-aminophenyl)butanoate (5)

To a solution of 4–(4-aminophenyl)butyric acid (1.0 g, 5.58 mmol) in methanol (40 ml), was added sulphuric acid (4 ml) and it was stirred at room temperature for 12 h under argon. The mixture was neutralised with 10% NaOH to pH 8, concentrated under reduced pressure and then extracted with ethyl acetate. The organic layer was dried over Na_2_SO_4_, filtered, concentrated under reduced pressure to afford compound **5** as yellow oil in 86% yield. R_f_ = 0.27 (3:7 ethyl acetate: hexanes). ^1^H NMR (500 MHz, MeOD) δ 6.96 (d, *J*=8.0 Hz, 2 H), 6.70 (dd, *J*=6.3 Hz, 2.0 Hz, 2 H), 3.67 (s, 3 H), 2.53 (t, *J*=7.0 Hz, 2 H), 2.32 (t, *J*=7.5 Hz, 2 H), 1.91–1.85 (m, 2 H). ^13 ^C NMR (125 MHz, MeOD) δ 175.8, 146.3, 132.5, 130.1, 116.9, 51.9, 35.2, 34.0, 28.0. ESI MS (*m/z*) 194.11 [M + 1]^+^.

#### 3.1.4. Methyl 4–(4-(diethylamino)phenyl)butanoate (6a)

A mixture of compound **5** (0.40 g, 2.07 mmol), iodoethane (0.42 ml, 5.17 mmol) and potassium carbonate (0.85 g, 6.21 mmol) in DMF was stirred at room temperature for 24 h under argon. The mixture was concentrated under reduced pressure, and then extracted with ethyl aceatate. The organic layer was washed with saturated NaHCO_3_ solution, dried over Na_2_SO_4_, concentrated under reduced pressure and purified by MPLC (Biotage SNAP HP-Sil column) to afford compound **6a** as white oil in 76% yield. R_f_ = 0.39 (1:9 ethyl acetate: hexanes). ^1^H NMR (500 MHz, CDCl_3_) δ 7.07 (d, *J*=8.5 Hz, 2 H), 6.67 (d, *J*=8.5 Hz, 2 H), 3.70 (s, 3H), 3.38–3.34 (*m*, 4 H), 2.59 (t, *J*=7.5 Hz, 2 H), 2.37 (t, *J*=7.5 Hz, 2 H), 1.99–1.93 (m, 2 H), 1.19 (t, *J*=7.5 Hz, 6 H). ^13 ^C NMR (125 MHz, CDCl_3_) δ 174.1, 146.2, 129.3, 128.2, 112.2, 51.4, 44.4, 34.1, 33.5, 26.9, 12.6.

#### 3.1.5. Methyl 4–(4-(dipropylamino)phenyl)butanoate (6b)

A mixture of compound **5** (0.36 g, 1.87 mmol), iodopropane (0.46 ml, 4.67 mmol) and potassium carbonate (0.78 g, 5.61 mmol) in DMF was stirred at room temperature for 24 h under argon. The mixture was concentrated under reduced pressure, and then extracted with ethyl aceatate. The organic layer was washed with saturated NaHCO_3_ solution, dried over Na_2_SO_4_, concentrated under reduced pressure and purified by MPLC (Biotage SNAP HP-Sil column) to afford compound **6b** as white oil in 76% yield. R_f_ = 0.38 (1:9 ethyl acetate: hexanes). ^1^H NMR (500 MHz, CDCl_3_) δ 7.03 (dd, *J*=11.5 Hz, 3.0 Hz, 2 H), 6.60 (dd, *J*=11.5 Hz, 2.0 Hz, 2 H), 3.68 (s, 3H), 3.22 (t, *J*=7.5 Hz, 4 H), 2.55 (t, *J*=7.5 Hz, 2 H), 2.35 (t, *J*=7.5 Hz, 2 H), 1.96–1.90 (m, 2H), 1.65–1.58 (m, 4H), 0.94 (t, *J*=7.5 Hz, 6 H). ^13 ^C NMR (125 MHz, CDCl_3_) δ 174.3, 146.7, 129.3, 127.9, 112.0, 53.1, 51.5, 34.1, 33.6, 26.9, 20.5, 11.6. ESI MS (*m/z*) 279.20 [M + 1]^+^.

#### 3.1.6. 4–(4-Aminophenyl)-N-hydroxybutanamide (7)

Hydroxylamine hydrochloride (0.95 g, 13.7 mmol) in methanol (4 ml) was added to a solution of potassium hydroxide (0.78 g, 13.7 mmol) in methanol (4 ml) at 0 °C. The mixture was stirred for 15 min at 0 °C and the precipitated potassium chloride was removed and the filtrate was used as such; To a solution of the compound **5** (0.13 g, 0.34 mmol) in tetrahydrofuran (4 ml) was added to freshly prepared hydroxylamine at 0 °C and stirred at the same temperature for 2 h. The mixture was neutralised with 3 N HCl to pH 7 and extracted with ethyl acetate. The organic layer was dried over Na_2_SO_4,_ concentrated under reduced pressure and purified by HPLC to afford compound **7** in 40% yield. R_f_ = 0.27 (9:1 ethyl acetate: methanol). ^1^H NMR (500 MHz, DMSO-D6) δ 10.33 (s, 1 H), 8.66 (s, 1 H), 6.81 (d, *J*=8.5 Hz, 2 H), δ 6.47 (dd, *J*=10.8 Hz, 2.0 Hz, 2 H), 4.80 (s, 2 H), 2.36 (t, *J*=7.5 Hz, 2 H), 1.92 (t, *J*=7.5 Hz, 2 H), 1.71–1.65 (m, 2H). ^13 ^C NMR (125 MHz, DMSO-D6) δ 169.6, 147.0, 129.2, 129.1, 114.5, 34.4, 32.4, 28.0. ESI MS (*m/z*) 195.11 [M + 1]^+^.

#### 3.1.7. 4–(4-(Diethylamino)phenyl)-N-hydroxybutanamide (8a)

Hydroxylamine hydrochloride (4.11 g, 59.19 mmol) in methanol (5 ml) was added to a solution of potassium hydroxide (3.32 g, 59.19 mmol) in methanol (5 ml) at 0 °C. The mixture was stirred for 15 min at 0 °C and the precipitated potassium chloride was removed and the filtrate was used as such; To a solution of the compound **6a** (0.33 g, 1.32 mmol) in tetrahydrofuran (10 ml) was added to freshly prepared hydroxylamine at 0 °C and stirred at the same temperature for 2 h. The mixture was neutralised with acetic acid to pH 7 and extracted with ethyl acetate. The organic layer was dried over Na_2_SO_4,_ concentrated under reduced pressure and purified by MPLC (Biotage SNAP KP-C18 column) to afford compound **8a** in 40% yield. R_f_ = 0.18 (3:2 ethyl acetate: hexanes). ^1^H NMR (500 MHz, CDCl_3_) δ 6.99 (d, *J*=8.0 Hz, 2 H), 6.62 (d, *J*=9.0 Hz, 2 H), 3.32–3.28 (m, 4 H), 2.50 (t, *J*=7.5 Hz, 2 H), 2.09 (s, 2H), 1.88 (t, *J*=7.0 Hz, 2 H), 1.13 (t, *J*=6.5 Hz, 6 H). ^13 ^C NMR (125 MHz, CDCl_3_) δ 171.9, 146.3, 129.4, 128.2, 112.5, 44.6, 34.0, 32.3, 27.2, 12.6. ESI MS (*m/z*) 251.17 [M + 1]^+^.

#### 3.1.8. 4–(4-(Dipropylamino)phenyl)-N-hydroxybutanamide (8 b)

Hydroxylamine hydrochloride (2.25 g, 32.44 mmol) in methanol (5 ml) was added to a solution of potassium hydroxide (1.82 g, 32.44 mmol) in methanol (5 ml) at 0 °C. The mixture was stirred for 15 min at 0 °C and the precipitated potassium chloride was removed and the filtrate was used as such; To a solution of the compound **6b** (0.20 g, 0.72 mmol) in tetrahydrofuran (10 ml) was added to freshly prepared hydroxylamine at 0 °C and stirred at the same temperature for 2 h. The mixture was neutralised with acetic acid to pH 7 and extracted with ethyl acetate. The organic layer was dried over Na_2_SO_4,_ concentrated under reduced pressure and purified by MPLC (Biotage SNAP KP-C18 column) to afford compound **8b** in 46% yield. R_f_ = 0.27 (6:4 ethyl acetate: hexanes). ^1^H NMR (500 MHz, CDCl_3_) δ 6.98 (d, *J*=8.5 Hz, 2 H), 6.57 (d, *J*=8.5 Hz, 2 H), 3.20 (t, *J*=7.5 Hz, 4 H), 2.50 (t, *J*=7.5 Hz, 2 H), 2.12–2.06 (m, 2 H), 1.92 (m, 2 H), 1.63–1.55 (m, 4 H), 0.92 (t, *J*=7.5 Hz, 6 H). ^13 ^C NMR (125 MHz, CDCl_3_) δ 172.0, 146.7, 129.3, 127.7, 112.1, 53.1, 33.9, 32.3, 27.2, 20.5, 11.6. ESI MS (*m/z*) 279.20 [M + 1]^+^.

### 3.2. Biology

#### 3.2.1. Materials

Dulbecco’s Modified Eagle’s medium (DMEM) with L-glutamine was purchased from GenDEPOT (Barker, TX, USA) and RPMI 1640 medium, fatal bovine serum (FBS), and penicillin/streptomycin were purchased from Gibco BRL (Gaithersburg, MD, USA). Antibodies specific for *α*-tubulin, Ac-*α*-tubulin, Histone H3, Ac-histone H3, PARP, caspase 3, cleaved caspase 8, *β*-actin HDAC1, and HDAC6 were purchased from Cell Signalling Technology (Boston, MA, USA). Rad52 antibody and goat anti-rabbit IgG horseradish peroxidase conjugate were purchased from Santa Cruz Biotechnology Inc. (Dallas, TX, USA). Cell Titre 96 Aqueous One Solution cell proliferation assay kit was purchased from Promega (Madison, WI, USA). Amersham ECL select Western blotting detection reagent was purchased from GE Healthcare (Waukesha, WI, USA). HDAC fluorogenic assay kits (HDAC1, HDAC3, HDAC6, and HDAC8) were purchased from BPS Bioscience (San Diego, CA, USA). OxiSelect™ Comet Assay Kit was purchased from Cell Biolabs, Inc (San Diego, CA, USA).

#### 3.2.2. Cell culture

MDA-MB-231 cells were grown in DMEM with L-glutamine supplemented with streptomycin (500 mg/mL), penicillin (100 units/mL), and 10% foetal bovine serum (FBS). HL-60, H1975, U937, A549, U266, MCF-7/ADR and A2780 cells were grown in RPMI 1640 with L-glutamine supplemented with streptomycin (500 mg/mL), penicillin (100 units/mL), and 10% FBS. Cells were grown to confluence in a humidified atmosphere (37 °C, 5% CO_2_).

#### 3.2.3. Cell proliferation assay

Cells were seeded at a clear 96-well plate, the medium volume was brought to 100 µL, and cells were allowed to attach overnight. Various concentrations of compounds (**1**, **3**, **7**, **8a**, **8b** or DMSO) were added to the wells. Cells were then incubated at 37 °C for 3 days. Cell viability was determined using the Promega Cell Titre 96 Aqueous One solution cell proliferation assay. Absorbance at 490 nm and 690 nm as reference wavelength was read on Tecan Infinite F200 Pro plate reader, and values were expressed as percent of absorbance from cells incubated in DMSO alone.

#### 3.2.4. Western blot

HL-60 cells were seeded in 50 mm culture dish (1 × 106 cells/dish), and allowed to attach overnight. Cells were then treated with the indicated concentrations of chlorambucil or chloram-HDi for 24 h. Cells were harvested in ice-cold lysis buffer (23 mM Tris-HCl pH 7.6, 130 mM NaCl, 1% NP-40, 1% sodium deoxycholate, 0.1% SDS) and 30 µg of lysate per lane was separated by SDS-PAGE, followed by transferring to a PVDF membrane (Bio-Rad, Hercules, USA). The membrane was blocked with 5% skim milk in TBST, and then incubated with the corresponding primary antibody (*α*-tubulin, Ac-*α*-tubulin, HDAC1, HDAC6, Histone H3, Ac-histone H3, PARP, caspase 3, cleaved caspase 8, Rad52, H2AX, γH2AX, or *β*-actin). After being treated with the secondary antibody coupled to horseradish peroxidase (Santa Cruz, CA, USA), proteins were visualised by ECL chemiluminescence according to the manufacturer’s instruction (GE healthcare, USA).

#### 3.2.5. Comet assay

Comet assay was performed using OxiSelect™ comet assay kit (Cell Biolabs Inc., San Diego, CA, USA). The alkaline electrophoresis was conducted to detect any single-stranded and double-stranded DNA breaks (SSBs and DSBs) according to the manufacturer’s instruction. HL-60 cells were seeded in 50 mm culture dish (1 × 106 cells/dish), and allowed to attach overnight. Cells were then incubated with DMSO, chlorambucil (10 µM) or chloram-HDi (10 µM) for 24 h. Cells were harvested into 15 ml falcon tube and washed with Dulbecco’s phosphate-buffered saline (DPBS). Cell suspension was mixed with molten agarose, 75 µL of the mixture was added to the slides, and dried at 4 °C for 15 min in dark. The embedded cells were treated with lysis buffer at 4 °C for 30 min in dark, which was then replaced with pre-chilled alkaline buffer and placed at 4˚C for 30 min. After replacing alkaline buffer with TBE buffer, the embedded cells were incubated for 5 min, twice. Finally, the samples were electrophoresed in a horizontal chamber to separate intact DNA from the damaged fractions and washed with deionised water. After being incubated in 70% ethanol for 5 min, the slides were dried, stained with DNA dye for 15 min at rt and visualised by invert microscope Eclipse-TiU (Nickon, Tokyo, Japan). DNA tail and DNA head were measured with Image J software.

#### 3.2.6. Cell cycle arrest

HL-60 cells were treated with the indicated concentrations of chlorambucil or chloram-HDi for 24 h. Cells were resuspended in 300 µL of PBS, treated with 700 µL of ethanol, and gently vortexed. Cells were then incubated at 4 °C for 2 h, washed with PBS, resuspended in 500 µL of PBS containing 50 µg/mL of propidium iodide and 1 µg/mL of Rnase A. After being incubated for an additional 30 min at rt in the dark, cells were analysed by FACS flow cytometer and BD CellQuest Pro software.

#### 3.2.7. Hdac assay

Enzymatic HDAC assaywas performed following manufacturer’s protocol (BPS Bioscience). Briefly, HDAC assay buffer (35 µL) was mixed with 5 ml of BSA (1 mg/mL) and 5 µL of HDAC substrate (200 µM) in 96-well black plate. 5 µL of HDAC enzyme (7 ng/µL) was added to the well, followed by various concentrations of compounds (5 µL) or SAHA (5 µL) as a positive control, and then the resulting mixture was incubated at 37 °C for 30 min. After the incubation, 50 µL of undiluted 2× HDAC developer was added to each well. After the mixture was incubated at rt for 15 min, fluorescence intensity was measured using a microplate reader at 360 nm excitation and 460 nm emission wavelengths.

### 3.3. Molecular docking

In silico docking of chloram-HDi with the 3 D coordinates of the X-ray crystal structure of HDAC6 (PDB code: 5EF7) was accomplished using the AutoDock 4.2 programme downloaded from the Molecular Graphics Laboratory of the Scripps Research Institute. In the docking experiments carried out, water was removed from the 3 D X-ray coordinates while Gasteiger charges were placed on the X-ray structures of HDAC6 along with chloram-HDi using tools from the AutoDock suite. A grid box centred on the substrate binding pocket of HDAC6 enzyme with definitions of 60 × 60 × 60 points and 0.375 Å spacing was chosen for ligand docking experiments. The docking parameters consisted of setting the population size to 150, the number of generations to 27,000, and the number of evaluations to 2,500,000, while the number of docking runs was set to 100 with a cut-off of 1 Å for the root-meansquare tolerance for the grouping of each docking run. The docking model of HDAC6 with chloram-HDi was depicted in Figure 8 and rendering of the picture was generated using PyMol software (DeLanoScientific).

### 3.4. Cytochrome P450 inhibition assay

The inhibitory potency of chloram-HDi was determined with cytochrome P450 assays in the absence and presence of chloram-HDi (final concentrations of 0–50 µM with acetonitrile concentration less than 0.5%) using pooled human liver microsomes (Xenotech H0630). All experiments were performed in duplicate. Phenacetin *O*-deethylase, tolbutamide 4-hydroxylase, omeprazole hydroxylase, dextromethorphan *O*-demethylase, and midazolam 10-hydroxylase activities were determined as probe activities for CYP1A2, CYP2C9, CYP2C19, CYP2D6, and CYP3A, respectively, using cocktail incubation and tandem mass spectrometry, as described previously^50^^,^^51^.

## 4. Conclusion

Although chlorambucil remains one of the front-line treatment of CLL and malignant lymphomas, the occurrence of drug resistance is still a major hurdle to the successful cancer treatment. Among the mechanisms of drug resistance, it has been suggested that the activation of DNA repair machinery such as homologous recombination (HR) plays a critical role in the drug resistance of chlorambucil^17^^,^^52^.

**Figure 9.**
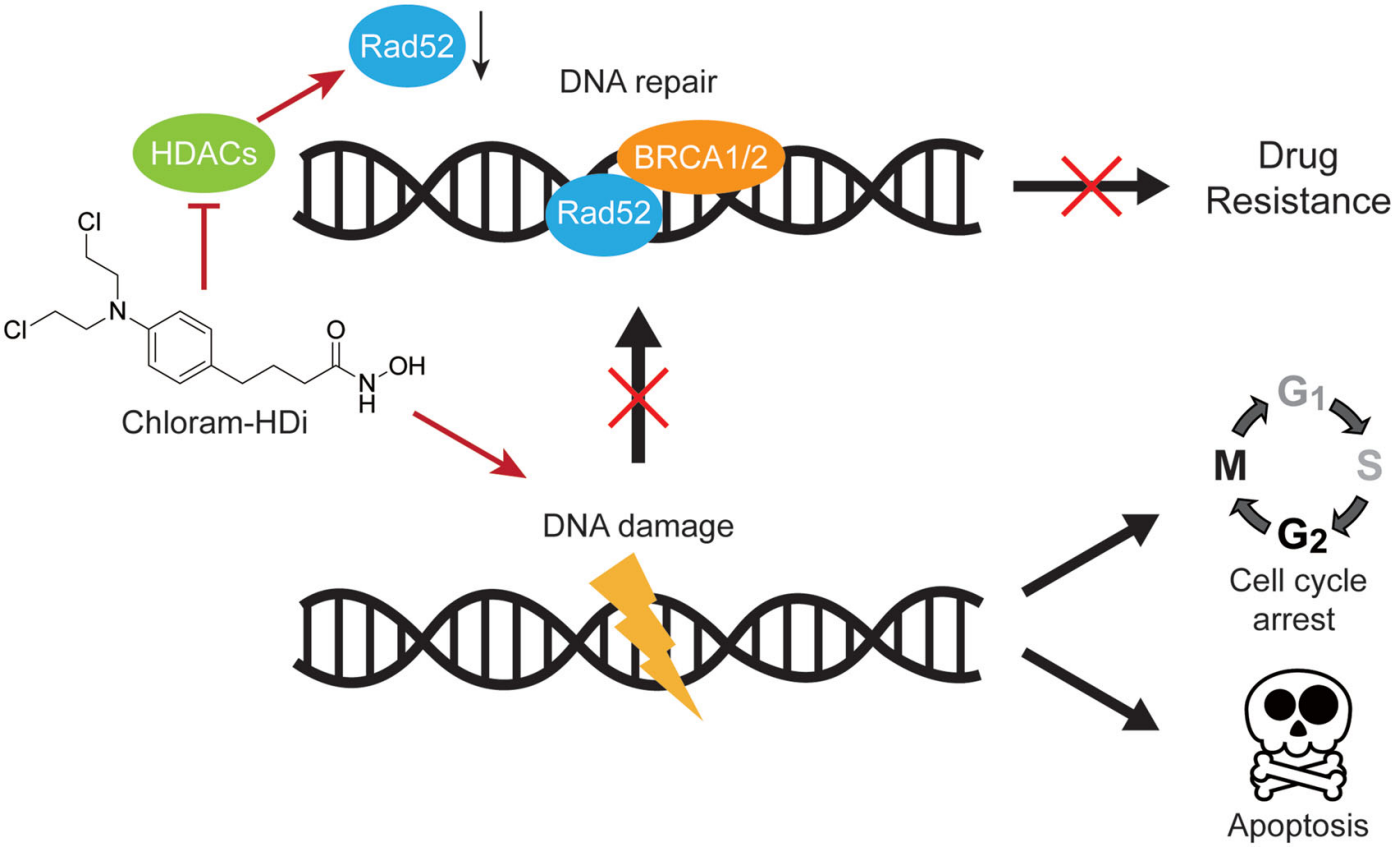
Mode of action of the hybrid molecule, chloram-HDi.

In this regard, we designed a novel hybrid molecule, chloram-HDi that simultaneously impaired DNA and HDAC enzymes (Figure 9). Chloram-HDi caused a remarkable DNA damage of HL-60 cells, in that % DNA content in the tail was xx% in comet assay and the DNA damage marker, *γ*-H2AX was significantly upregulated in western blot analysis. Chloram-HDi also caused the reduction of the DNA repair protein, Rad52, magnifying its DNA-damaging effect by disrupting Rad52-mediated homologous recombination (HR). Furthermore, chloram-HDi inhibited HDAC enzymes to induce the acetylation of *α*-tubulin and histone H3. It has been reported that the inhibition of HDACs induces the acetylation of Hsp90, which impairs the chaperone activity of Hsp90 and consequently causes the depletion of its client protein, Rad52^53^. As a result, chloram-HDi efficiently promoted the cell cycle arrest at the G_2_/M phases and resulted in apoptotic cell death, evidenced by the cleavage of PARP, caspase 3, and caspase 8. Overall, these findings firmly supported that chloram-HDi could serve as a potential drug candidate for the treatment of leukaemia, warranting further studies on *in vivo* evaluation as well as ADMET profiles. These studies will be reported in due course.
